# Implementation of Passing Vehicle Search Algorithm for Optimization of WEDM Process of Nickel-Based Superalloy Waspaloy

**DOI:** 10.3390/nano12244394

**Published:** 2022-12-09

**Authors:** Rakesh Chaudhari, Izaro Ayesta, Mikesh Doshi, Sakshum Khanna, Vivek K. Patel, Jay Vora, Luis Norberto López de Lacalle

**Affiliations:** 1 Department of Mechanical Engineering, School of Technology, Pandit Deendayal Energy University, Gandhinagar 382007, India; 2 Department of Mechanical Engineering, Escuela Superior de Ingenieros, University of the Basque Country, Alameda de Urquijo s/n., 48013 Bilbao, Spain; 3 School of Technology, Pandit Deendayal Energy University, Raisan, Gandhinagar 382007, India

**Keywords:** nickel-based superalloy, Waspaloy, passing vehicle search algorithm, wire electrical discharge machining (WEDM), MWCNTs

## Abstract

Nickel-based superalloys find their main use in missile engines, atomic devices, investigational aircraft, aerospace engineering, industrial applications, and automotive gas turbines, spacecraft petrochemical tools, steam power, submarines, and broader heating applications. These superalloys impose certain difficulties during the process fabrication owing to their levels of higher hardness. In the current study, the precise machining of Waspaloy was attempted through the wire electrical discharge machining (WEDM) technique. A multi-objective optimization has been performed, and the influence of multi-walled carbon nanotubes (MWCNTs) has been assessed using the passing vehicle search (PVS) algorithm. The effects of machining variables like current, T_off_, and T_on_ were studied using the output measures of material removal rate (MRR), recast layer thickness (RLT), and surface roughness (SR). The Box–Behnken design was applied to generate the experimental matrix. Empirical models were generated which show the interrelationship among the process variables and output measures. The analysis of variance (ANOVA) method was used to check the adequacy, and suitability of the models and to understand the significance of the parameters. The PVS technique was executed for the optimization of MRR, SR, and RLT. Pareto fronts were derived which gives a choice to the user to select any point on the front as per the requirement. To enhance the machining performance, MWCNTs mixed dielectric fluid was utilized, and the effect of these MWCNTs was also analyzed on the surface defects. The use of MWCNTs at 1 g/L enhanced the performance of MRR, SR, and RLT by 65.70%, 50.68%, and 40.96%, respectively. Also, the addition of MWCNTs has shown that the machined surface largely reduces the surface defects.

## 1. Introduction

The introduction of high-strength superalloys, combined with the constant adaptation of industrial developments to enable the production of premium-quality mechanisms at minimal built-up expenses, minimal rejection rates, and higher built-up rates is one of the ultimate challenges for contemporary aerospace engineering [1,2]. Superalloys largely impose several critical concerns regarding the surface morphology behaviour [3]. These components are built-up under the accurate standards of the aerospace industry [4]. Most of the materials used in aircraft engine design, specifically in gas turbines, are nickel-based superalloys [5,6]. Nickel-based superalloys serve a vital role in gas turbine engines. Such components can be structured as single crystals, totally removing all borders of the high angle grains, or they can comprise either equiaxed grains or columnar grains [7,8]. These superalloys find their use primarily in missile engines, atomic devices, investigational aircraft, aerospace applications, industrial processes, and automotive gas turbines, spacecraft petrochemical tools, steam power, submarines, and other heating applications [9,10,11]. Nickel-based superalloys impose certain difficulties during the fabrication owing to their higher hardness [12,13]. Nickel-based superalloys, including Waspaloy, are difficult to cut due to the higher strength and hardness, poor chip formations, lower heat conductivity, work hardening at elevated temperatures [14,15,16]. Waspaloy is a nickel-based superalloy and belongs to an exclusive family of metallic materials, with an exclusive combination of extreme temperature resistance, hardness, and opposition to deterioration in corrosive conditions, such as nuclear power plants and chemical processing facilities [17,18]. Waspaloy is mostly used in segments that necessitate extreme strength and decent corrosion resistance at high temperatures, such as in heated turbine parts [19,20]. The highest service temperature of Waspaloy is up to 750 °C [17]. However, the presence of an extreme cobalt particle slightly increases the cost of the mixture. Waspaloy has a face-targeted three-dimensional austenitic (γ) medium and is hardened especially via the means of submicron precipitates of gamma-mark (γ′), which make up 25% via the means of the quantity inside the completely hardened material [17]. The machining of nickel-based alloys such as Waspaloy imposes several difficulties owing to higher tool wear and breakage, and unwanted machined surfaces [21]. The strength is largely retained during processing which increases the cutting strength. The heat obtained while machining Waspaloy is not dissipated through chip, which leads to rapid tool wear [22,23]. Low thermal conductivity at the chip/tool interface generated a strong temperature gradient, which further accelerated the tool failure [23]. Cutting tools often result in unwanted chips and poor surface quality [24]. Traditional machining of Wasploy further leads to the rough machined surface, improper dimensional correctness, and difficulty in producing the complex shape geometries [25,26,27]. The dimension accuracy of conventional machining is lower compared to non-conventional machining. Thus, Waspaloy is most suitable to being machined via non-traditional machining techniques.

Wire electrical discharge machining (WEDM) is a process of removing material from a base metal using thermal energy [28,29,30]. Unlike laser cutting, the WEDM process does not require machine-driven forces in the elimination process. Electrical discharge machining (EDM) has shown improved performance in situations involving materials with high strength and high hardness, as well as in obtaining intricate forms [31,32]. The material removal mechanism involves the melting and vaporization of work material with an excessive temperature spark [33]. This imposes different defects on the machined surfaces, such as those of crack formation, porosity, residual stress, increased surface roughness (SR), and development of additional recast layer thickness (RLT) [34,35]. It becomes essential to use appropriate ways to eliminate the damaged surface layer and re-establish surface characteristics [36]. The rate of material erosion in non-conventional processes, including WEDM, is challenging, in addition to impacting surface integrity aspects [37]. As a result, improving material removal rate (MRR), and damaged surface layer, and surface discontinuities will be highly preferred. All these objectives can be achieved by using suitable type and amount of nano-powder in the dielectric fluid [38,39]. Various nano-powders like Si, Al_2_O_3_, Al, CNT, Cr, MWCNT, etc. were used by researchers to enhance the output measures of EDM [40,41]. During the process, various features of nano-powders like conductivity, size, amount etc. play important roles in improving material performances [42,43]. Excellent features of carbon nanotubes (CNTs), such as higher electrical and mechanical properties, enhance the machining performance and surface quality are always preferable [44,45]. When CNTs are combined with a dielectric fluid, their unique features, like large thermal conductivity and enlarged strength, can affect the thermo-electrical properties of the dielectric medium, potentially improving EDM performance [46]. CNTs are composed of various forms, such as single-walled CNTs (SWCNT) and multi-walled CNTs (MWCNT). SWCNTs resemble seamless cylinders, each with a single layer of graphene, while MWCNTs are made up of numerous concentric graphene layers in a cylindrical form. MWCNTs are also more thermally and chemically stable than SWCNTs [46]. Furthermore, because MWCNTs can be mass-manufactured, their processing costs are lower than those of SWCNTs. Furthermore, MWCNTs have stronger intensity and corrosion resistance and are less prone to oxide formation than SWCNTs [47].

Misra et al. [48] used the WEDM machining process for obtaining the higher MRR and lower TWR values for the Waspaloy. Their results depicted that their selected machining variables such as servo feed, wire feed, and wire tension has not shown any improvement in MRR. Further, they empathize that selection of other variables like T_on_, T_off_, SV, and current could improve the machining performance of Waspaloy. Kumara et al. [31] studied effect of WEDM parameters of T_on_, T_off_, wire feed, and wire tension by performing experiments at three distinct levels on MRR, and SR. To show the effect of these parameters, expected records were utilized to determine the parametric effect in the form of graphical depictions. The predicted data from the generated models were utilized to obtain suitable combination of machining variables for the required outputs. The ideal value was validated in comparison to the expected value. Singh and Garg [49] investigated the outcome of WEDM variables of T_on_, T_off_, SV, current (IP), and wire tension on MRR for hot die steel. Their obtained results have shown that MRR was improved, with increased value of T_on_ and current, and decreased value of T_off_ and SV. Kumar et al. [50] investigated the impact of WEDM variables on the machinability of a Nimonic-90 superalloy. The effects of current, T_on_, T_off_, SV, and WF on Nimonic-90 have been examined in terms of cutting speed. Obtained results have shown that current, T_on_, and T_off_ had substantial impacts on the improvement in cutting speed. Soni et al. [51] used a nickel-based superalloy for enhancing the performance of MRR and SR using the WEDM method. An increment in T_on_, combined with a drop in T_off_ and SV, has obtained improvement in both MRR and SR. Their results depicted that T_on_, lower than 125 µs and SV greater than 20 V, will be a handful for the reduction of micro-crack and RLT formation. Manjaiah et al. [52] used the design of the experimental plan and studied the machining characteristics such as MRR, SR, and surface morphology for nickel-based superalloys. Their obtained results have shown that T_on_, SV, and T_off_, had the most significant influence in affecting MRR and SR. Shabgard and Behnam [53] used CNTs, combined with a dielectric medium, to increase the machining capability of the Ti6Al4V alloy using copper electrodes. SEM images has shown that the presence of CNT particles in the dielectric fluid decreased the machined surface micro-cracks. Also, the addition of CNTs into the dielectric fluid provided significant benefits in machining stability, resulting in lower TWR. The use of CNTs has also reduced SR by reducing spark energy and distributing it uniformly. Izman et al. [54] investigated the effect of kerosene dielectric with and without the addition of MWCNTs for EDM performance. The impacts of three controlled variables were analyzed, namely T_on_, spark interval, and peak current. When compared to performance dielectric without powder of EDM process, the addition of MWCNTs resulted in a significant increase in quality in MRR (7%) and SR (9%) as well as a reduction in RLT. Another study reported by S. Mai et al. [55] used CNTs to float and dissolve homogeneously in the dielectric fluid in EDM process. The effect of using CNTs on machining efficiency and surface integrity was investigated. The process of chemical vapor deposition was used to create the CNTs, and used at a 0.4 g/L amount with dielectric fluid. A substantial drop in RLT and surface features was clearly evident through their obtained findings. The process variables were used as 1 A current, 2 µs pulse duration, 280 V open-circuit voltage, and 70 V gap voltage. Improvements of 70% and 66% were also recorded in SR and machining time, respectively. They revealed that it was due to the nanoscale properties of CNTs. The electric arcs were more evenly distributed and uniform throughout the electrical potential gap, considerably improving the performance. Carbon nanotubes were projected to be employed in a variety of EDM applications.

Studies from past researchers show that the multi-objective optimization of Waspaloy has not been explored to its fully potential, and neither has the influence of MWCNTs on nickel-based superalloys. Indeed, as per the authors’ information, multi-objective optimization and the effect of MWCNTs on Waspaloy for WEDM variables were not investigated using the passing vehicle search (PVS) algorithm. In the present work, the aforesaid research gap was narrowed, and a study pertaining to the machinability of Waspaloy was employed through the WEDM process. In the present work, T_on_, T_off_, and current were selected as the machining parameters, while MRR, SR, and RLT were chosen as the response variables. A Box–Behnken design was applied to generate the experimental matrix. The analysis of variance (ANOVA) method was employed to assess the suitability and importance of the parameters. The PVS technique was executed for the optimization of MRR, SR, and RLT. In the last section, MWCNT nano-powders were mixed with a suitable concentration of the dielectric fluid to eliminate the damaged surface layer and re-establish surface characteristics. Lastly, an examination of MWCNTs on machined surfaces was studied through scanning electron microscopy (SEM). The authors believe that results of the present study will be helpful for researchers and industrial users for their upcoming works.

## 2. Materials and Methods

### 2.1. Prepartion of MWCNTs

The multiwall carbon nanotubes (MWCNTs) were synthesized using the modified hydrothermal process, as per our reported work [56]. The key components were required in synthesizing MWCNT NaOH, anhydrous C_2_H_5_OH, and PEG. All the chemicals were purchased from Sigma Aldrich Inc. along with DI water of 18.2 mΩ cm and used without any purification. The MWCNTs were prepared by adding 4 g NaOH and 2 g PEG into a mixture of ethanol and water (8:1) and vigorously stirred for 75 min. After the mixture was stirred, it was transferred to a hydrothermal autoclave. The experiments were carried out in the temperature range from 120–200 °C with 40 °C interval overnight in a furnace and then naturally cooled down in 6 h to room temperature. The resultant material was centrifuged and washed 3–4 times with ethanol and water. Later, the as-synthesized materials were dried at 75 °C for few hours. The morphological and structural studies of synthesized samples were carried out under field emission scanning electron microscope (FESEM) (Carl Zeiss, ULTRA 55, Bangalore, India), X-ray diffraction spectroscopy (Panalytical, λ = 1.54) and micro-Raman spectroscopy conditions (Renishaw in via Raman Microscope, Pune, India).

### 2.2. Eperimental Plan

**Figure 1:**
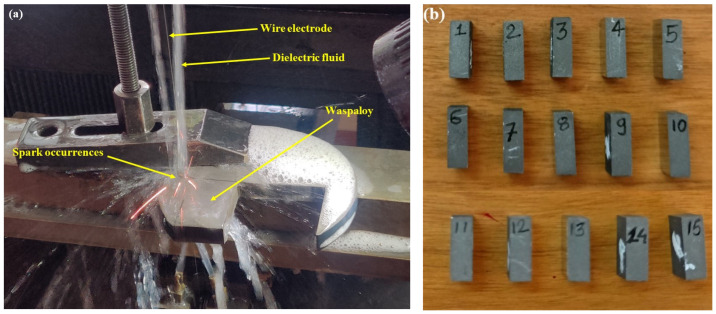
(**a**) Experimental setup of WEDM process (**b**) cut specimens as per BBD approach.

Herein, the experiments were performed on the WEDM process by using Waspaloy as the work material and molybdenum as an electrode with a diameter of 0.18 mm. Table 1 displays the composition of the selected work material. The work material Waspaloy has a density of 8.19 g/cc. Figure 1a shows experimental setup employed in the present work. In the present study, machining variables (T_on_, T_off,_ and current) along with their levels were chosen on basis of device capacity, the studied literature for nickel-based alloys, and some preliminary experimental trials. The selected parameters at three levels are mentioned in Table 2. The RSM approach of the Box–Behnken design has been used to create an experimental plan with an aim to minimize the number of trials [57,58]. RSM gives mathematical relationships between machining parameters and output responses. Machining specimens of dimensions 15 mm × 10 mm × 5 mm were cut using the WEDM process. Figure 1b displays the cut specimens following the BBD matrix.

Equation (1) depicted the method of evaluation of MRR in (mm^3^/sec) by means by recording weight of machined specimens.

(1) $$\mathrm{MRR}=\frac{(W_{\mathrm{bm}}-{\;W}_{\mathrm{am}})\times 1000\;}{\rho \;\times \;t}$$

where W_bm_ and W_am_ are the weight of components in gram, ‘ρ’ is the density of the workpiece, and ‘t’ is time in second.

**Figure 2:**
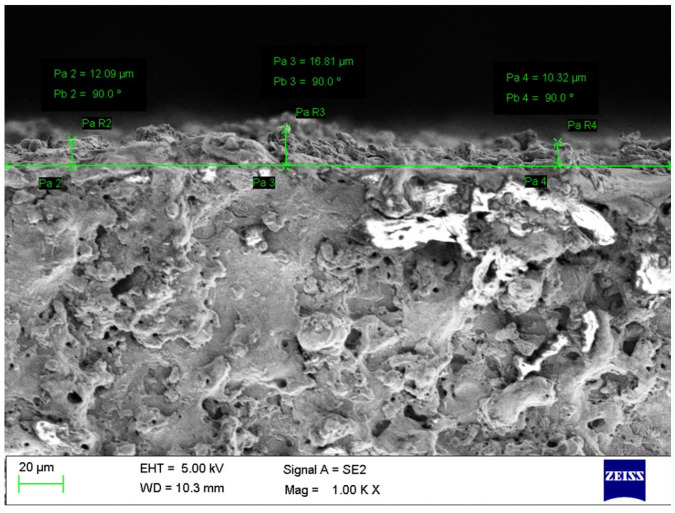
SEM image for measurement of RLT of experimental trial 1.

For precise applications, lower SR of the machined surface is must requirement. The nano-graphene powder-mixed WEDM process was employed to enhance the SR. The Surftest SJ-410 profilometer (Mitutoyo, Ahmedabad, India) was employed to record the value of SR. SR was recorded at multiple locations for every specimen and their average value was considered for analysis. SEM was employed for the determination of the RLT response. RLT was measured at multiple locations, and an average value was considered for analysis purpose. Figure 2 shows the SEM image for the measurement of the RLT for experimental run 1. Also, an examination of MWCNTs on machined surfaces was also studied. To understand the influence of MWCNTs on MRR, SR, and RLT, 1 g/L MWCNTs amount has been added to the dielectric fluid. SEM was utilized to assess surface defects formed on the machined surfaces of conventional WEDM and nano-powder-mixed WEDM.

### 2.3. PVS Algorithm

Savsani and Savsani [59] have developed a novel technique of using the PVS algorithm. This technique has shown largely effective and efficient for multiple applications. The PVS algorithm consists of the mechanism which takes into the account of passing the vehicle on a two-lane highway. During its execution, one of the key principles of safe overtaking occasion (passing) has been taken into account. The execution of PVS method is reliant on multiple complex inter-reliant factors such as speed of vehicles, acceleration, overtaking capabilities of driver, space availability in traffic for overtaking the other vehicles, driver skills, traffic conditions, road conditions and weather situation. This technique consists of three forms of vehicles for the passing mechanism. These three types consist of front vehicle (FV), back vehicle (BV), and oncoming vehicle (OV). Consider a scenario where BV needs to overtake (pass) FV. This will be possible only if speed of BV becomes higher than that of FV. Apart from this, some other factors such as position and speed of OV, space among them with changes in their speeds needs to be in favor for passing the BV over FV. Suppose that three types of vehicles like BV, FV, and OV have velocities of V_1_, V_2_, and V_3_, respectively, while passing on a two-lane highway. For a particular time instance, assume that x is the distance among the FV and BV, and y is the distance among OV and FV. There are two key possibilities in accounting for the velocities of FV and BV. The passing of one vehicle will take place if any of the vehicle is having lower velocity in comparison with the other. Also, another condition needs to be satisfied if the distance from FV at which passing occurs is less than the distance covered by OV. This shows that various conditions arise for the selected vehicles for passing. The mathematical formulation was utilized for these conditions to solve multiple objectives.

## 3. Results and Discussions

The WEDM parameters, along with the evaluated response values of MRR, SR, and RLT, are displayed in Table 3. Minitab v17 was used to analyze the obtained results. With the use of obtained results, non-linear regression equations were derived through RSM approach and Minitab software. Analysis of variance (ANOVA) was employed to determine the suitability and importance of the parameters. For ANOVA, 95% of CI was considered, which means *p* value lowers than 0.05 depict the impact of selected variables to affect the selected response [60,61]. The influence of the machining variables on responses was then investigated, using main effect graphs for individual regression models.

### 3.1. Analysis of MWCNT

**Figure 3:**
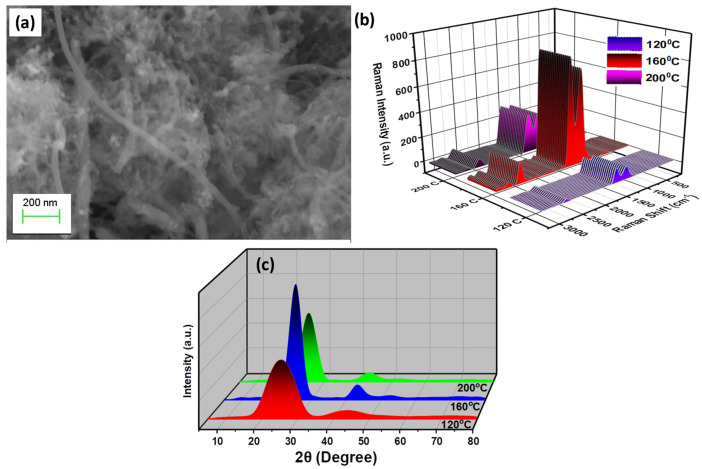
MWCNT analysis: (**a**) SEM of MWCNT nano-powder (**b**) Raman profile (**c**) XRD spectra of MWCNT [62].

The morphological studies of MWCNTs were carried out using SEM, as shown in Figure 3a. The figure’s images confirm the formation of the elongated and even tubular nanostructures of carbon, structures which are shaped like nanotubes. The FESEM also provide and inside to various nanotubular structures, having average length and diameter of around 10 ± 5 μm and 20 ± 10 nm, respectively. Further, the structural analysis of various MWCNTs fabricated at different temperatures was carried out using Raman spectroscopy, as shown in Figure 3b. In the synthesized MWCNT samples, graphitic bands were observed to have a distinct peak and shoulder peak at 1590 cm^−1^ and 1764 cm^−1^, respectively, indicating the presence of a G band or C-C bond. Another peak at 1361 cm^−1^ was also observed, which can be associated with a disorder in carbon systems known as the D band. Further, the peak at 2695 cm^−1^ is the D band’s overtone, termed a G band. The highest intensity was observed at 160 °C when comparing the sample hydrothermal to various temperatures, indicating complete MWCNT production. The optimal temperature of 160 °C for 24 h was employed for the subsequent treatment and characterization of the fabrication of MWCNT.

Figure 3c shows the X-ray diffraction spectroscopy for entire MWCNTs produced at various temperatures. The peaks at 78.19°, 55.42°, and 43.18° correspond to the (101), (004), (100), and (002) planes, respectively (JCPDS No. 23–64) [56]. On increasing the temperature, 160 °C, more disordered phases of MWCNT formed, leading to a reduction in peak intensity and boarding. Thus, the results were found to be in good agreement with the reported literature, suggesting the formation of wurtzite-structured cylindrical carbon tubes.

### 3.2. Mathematical Regression Equations and ANOVA of MRR, SR, and RLT

By using the obtained results of all responses, non-linear regression equations were derived from RSM approach through Minitab software. Equations (2)–(4) shows the obtained regression equations by using the backward elimination method for MRR, SR, and RLT, respectively. Non-significant variables were removed from the statistical model since they had no meaningful influence on the test value.

(2) $$\begin{array}{c} \mathrm{MRR}\;=2.421+0.026\cdot \mathrm{Current}-0.04535\cdot T_{\mathrm{off}}-0.02530\cdot T_{\mathrm{on}\;}+0.02000\\ \;\;\;\;\;\;\cdot \mathrm{Current}\cdot \mathrm{Current}+0.000389\cdot {\;T}_{\mathrm{off}}\cdot T_{\mathrm{off}\;}+0.000219\cdot {\;T}_{\mathrm{on}}\\ \;\;\;\;\;\;\cdot T_{\mathrm{on}\;}+0.000472\cdot {\;T}_{\mathrm{off}}\cdot T_{\mathrm{on}\;} \end{array}$$

(3) $$\begin{array}{c} \mathrm{SR}\;=5.889-0.630\cdot \mathrm{Current}-0.0994\cdot T_{\mathrm{off}}-0.0041\cdot T_{\mathrm{on}\;}+0.0662\cdot \mathrm{Current}\\ \;\;\;\;\;\;\cdot \mathrm{Current}+0.000405\cdot {\;T}_{\mathrm{on}}\cdot T_{\mathrm{on}\;}+0.01781\cdot \;\mathrm{Current}\cdot T_{\mathrm{on}\;} \end{array}$$

(4) $$\begin{array}{c} \mathrm{RLT}\;=16.30+0.526\cdot \mathrm{Current}-0.4122\cdot T_{\mathrm{off}}-0.1810\cdot T_{\mathrm{on}\;}+0.1232\\ \;\;\;\;\;\;\cdot \mathrm{Current}\cdot \mathrm{Current}+0.00930\cdot {\;T}_{\mathrm{off}}\cdot T_{\mathrm{off}\;}+0.001388\cdot {\;T}_{\mathrm{on}}\\ \;\;\;\;\;\;\cdot T_{\mathrm{on}\;}-0.0297\cdot \;\mathrm{Current}\cdot T_{\mathrm{off}\;}+0.0050750\cdot {\;T}_{\mathrm{off}}\cdot T_{\mathrm{on}\;} \end{array}$$

Analysis of variance (ANOVA) has been employed for statistical analysis to confirm the suitability and importance of their parameters. For ANOVA, 95% of CI was considered, which means that a *p* value lower than 0.05 depicts the impact of selected variables in affecting the selected response. 

Statistical analysis of MRR through ANOVA was depicted in Table 4. It indicates that the derived model is significant for MRR. The linear interaction model is significant with all the parameters of current, T_off_, and T_on_. The square interaction model was found to obtain substantial outcome on the interaction of T_on_ × T_on_. The other square interactions were not seen to have any substantial impact on MRR. In terms of 2-way interaction, T_off_ × T_on_ was found to have significance in addition to the model term. In addition to the model terms, lack-of-fit was found to be non-significant which shows the suitability of developed model term to forecast the future outcomes [58]. Competence of the suggested regression was determined through R^2^ values. Minor difference between R^2^ values showed the suitability of the obtained results and the proposed model [63].

Table 5 shows the statistical analysis of SR using the ANOVA technique. It shows that obtained model is significant for SR. The linear interaction model is significant with all the parameters of current, T_off_, and T_on_. The model terms indicate that the obtained model for SR was significant. The linear interaction model was also observed to have significance, including the parameters of current, T_off_, and T_on_. The square interaction model and 2-way interaction model were also observed to have a significant. This includes the interaction terms of T_on_ × T_on_ and current × T_off_. In addition to the model terms, the lack of fit was found to be non-significant, which shows the suitability of the developed model terms to forecast the future outcomes. Competence of the suggested regression was determined through R^2^ values. Minor difference between R^2^ values showed the suitability of the obtained results and the proposed model.

Table 6 showed the ANOVA of RLT. The model term indicates that the obtained model for RLT was significant. The linear interaction model was also observed to have significance, including the parameters of current and T_on_. A higher *p* value of 0.908 for T_off_ suggests that it does not reflect any significant effect on the RLT response. The square interaction model and 2-way interaction model were also observed to have a significant effect. This includes the interaction terms of T_off_ × T_off_, T_on_ × T_on_, current × T_off_, and T_off_ × T_on_. In addition to the model terms, lack-of-fit was found to be non-significant, showing the suitability of developed model terms to forecast the future outcomes. The competence of the suggested regression was determined through R^2^ values. Minor differences between R^2^ values showed the suitability of the obtained results and the proposed model.

### 3.3. Residual Plots

**Figure 4:**
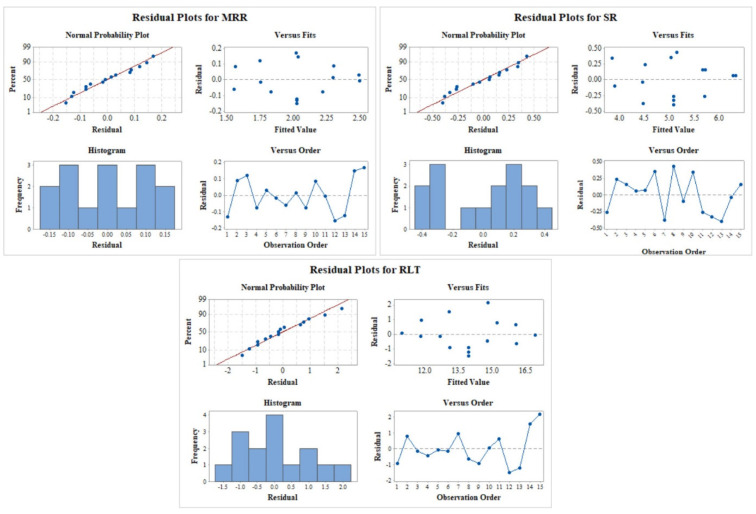
Residual plot for MRR, SR, and RLT.

Figure 4 displays the residual plots for the output measures of the process. The confirmation of residuals suggests the satisfactory outcomes of ANOVA findings. The normality plot validates that complete residuals are on the straight line. It specifies the correct assumptions of the model, and also signifies the normal distribution of errors [64]. Figure 4 validated the outcome of the derived results, which in turn validated the results in case of all the performance measures. For the residual versus fitted plot in Figure 4 for all responses, the obtained residuals are found to be randomly adjacent to both sides of the line, which shows the verification of better statistical analysis for ANOVA of the response [65]. The non-appearance of a significant pattern accomplished the crucial necessity of significant ANOVA in residual versus observation orders plot [65]. Therefore, the derived outcomes from residuals have shown good ANOVA findings and satisfy the required conditions. 

### 3.4. Contour Plots

**Figure 5:**
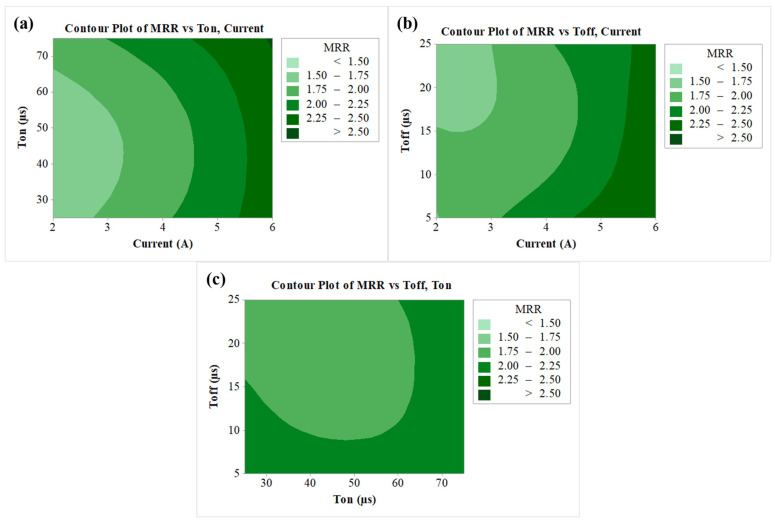
Contour plots for (**a**) MRR vs T_on_, current, (**b**) MRR vs T_off_, current, (**c**) MRR vs T_off_, T_on_.

An analysis of the output variable for MRR has been carried out by considering the variation in two input variables by using the contour plot. The contour plot of MRR is shown in Figure 5a–c, wherein two input variables were varied by keeping the third parameter at a constant level. Different colour codes were used to see the changes in the response value. As per Figure 5a, when the value of current increases, MRR also increases, an effect which can be observed from the different colours. During the process, discharged energy is converted into thermal energy [66]. The rise in thermal energy consequently melts and vaporizes the work material. Thus, the increment in current enlarges the thermal energy and then subsequently escalates the MRR [67]. When current was at maximum level of 6 A and at any value of T_on_, maximum MRR was obtained (>2.50 g/min). At the lower value of current around 2 A, the lowest MRR was obtained (<0.15 g/min). A similar effect can be observed from Figure 5b for MRR vs T_off_, current plot, wherein the maximum value was obtained at a higher current of 6 A and at all values of T_off_ (between 5 µs and 25 µs). MRR values in between 2.25 to 2.5 g/min were depicted in the multiple range of parameters from the plot of MRR vs T_off_, T_on_, as per Figure 5c. This includes the combination of lower T_off_ at any value of T_on_ and, also, higher T_on_ at any value of T_off_.

**Figure 6:**
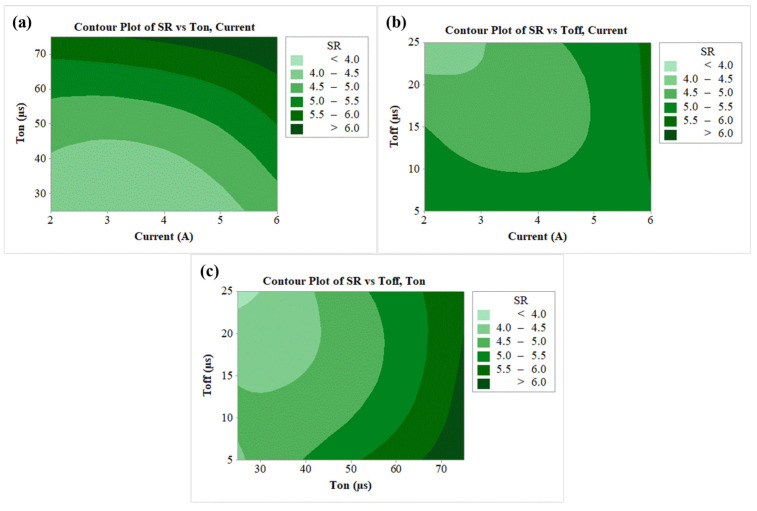
Contour plots for (**a**) SR vs T_on_, current, (**b**) SR vs T_off_, current, (**c**) SR vs T_off_, T_on_.

A similar analysis from the contour plot was carried out for SR as per the results shown in Figure 6a–c. As per the Figure 6a, the least SR (<4 µm) can be seen at the lower T_on_ and for current in the range of 2 A to 5 A. This was a result of a reduction in T_on_ which also reduced the discharge energy, thereby reducing the thermal energy of the sparks [68]. Lower current reduces the thermal energy and higher T_off_ decreases the active sparks [69]. Due to this reason, contour plot 6b shows the SR as less than 4 µm at the lowest current and highest T_off_. The contour plot of SR vs T_off_, as per Figure 6c, has shown the least SR at lowest T_on_ (25 µs) and highest T_off_ (25 µs).

**Figure 7:**
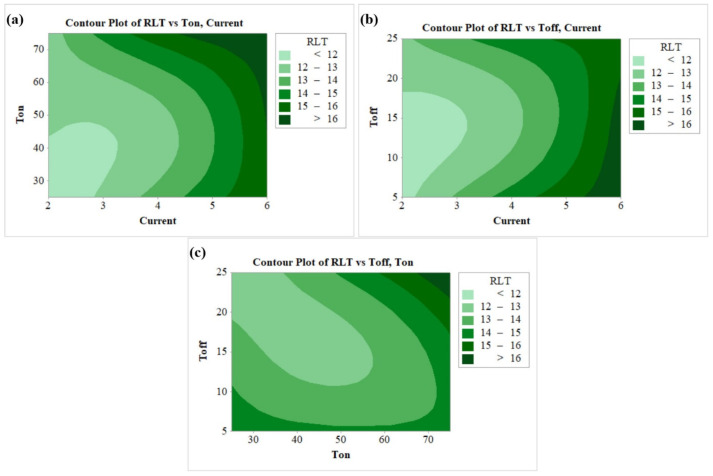
Contour plots for (**a**) RLT vs T_on_, current, (**b**) RLT vs T_off_, current, (**c**) RLT vs T_off_, T_on_.

Figure 7a–c shows the contour plots of RLT. With an increased amount of current, larger amounts of heat shifted to the work surface, thereby increasing the quantity of the molten metal. This molten metal then forms a recast layer on the machined surface [70]. The contour plot of Figure 7a for RLT vs T_off_, Current shows that RLT increases with rise in current and maximum RLT (>16 µm) was observed at highest current of 6 A and for T_on_ in the range of 50 µs to 90 µs. A similar conclusion can be obtained from Figure 7b, wherein the lowest RLT was found at the least current value of 2 A and for T_off_ in the range between 5 µs to 15 µs. Lower RLT in the range of 12–13 µm was depicted in the wide range of parameters from the plot of RLT vs T_off_, T_on_ as per Figure 7c. It shows a combination for the mentioned RLT as T_on_ up to 40 µs, and T_on_ in between 20 µs to 25 µs.

### 3.5. Effect of Machining Variables on Responses

**Figure 8:**
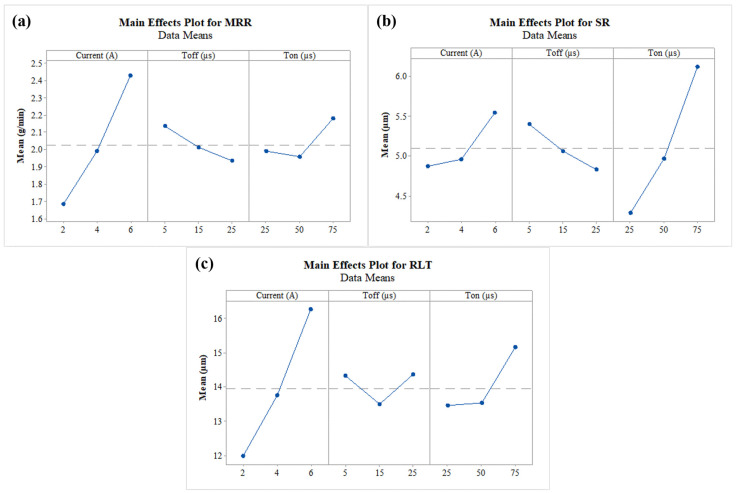
Main effect plot for (**a**) MRR, (**b**) SR, and (**c**) RLT.

Figure 8a–c demonstrates the impact of WEDM variables on output measures of MRR, SR, and RLT, respectively. In Figure 8a, influence of current, T_off_, and T_on_ was studied for MRR. It shows that MRR is enhanced by the rise in current and T_on_. The increases in the current and T_on_ enhance the discharge energy during the sparking process [71]. During the process, discharge energy is converted into thermal energy. The rise in thermal energy consequently melts and vaporizes the work material [72]. Thus, increment in both current and T_on_ enlarges the thermal energy and then subsequently escalates the MRR. Conversely, in case of the plot for MRR vs T_off_, a drop in MRR response was recorded. This was due to the fact that enhancement in the T_off_ results in the drop in discharge energy during the sparking process [73]. Thus, at higher T_off_ values, lower levels of thermal energy are produced. Owing to this, a drop in MRR can be seen with an enlargement in the input factor of T_off_.

The influence of current, T_off_, and T_on_ on SR can be seen in Figure 8b. During the erosion of the work material, larger number of craters is produced on the machined surface, which further affects the surface quality of the machined specimen [74]. Discharge energy generated during the process creates a larger amount of debris. This further generates deeper and wider craters on the machined surface specimens, and also depreciates the work surface [75]. As a result, the surface layer becomes rough, increasing the SR. As per Figure 8b, current and T_on_ had negative effects on the surface quality. An increase in the current and T_on_ increases the thermal energy released during the sparking process, which further escalates the SR [37]. The graph depicted decreased SR, with a rise in T_off_ owing to the development of small craters. Enhancement in the T_off_ value results in the drop in discharge energy during the sparking process [40]. Due to this lower energy, small craters on machined surface SR value are reduced by giving more favourable surface.

In Figure 8c, influence of current, T_off_, and T_on_ was studied for RLT. A substantial increment in RLT of the surface can be observed with intensification in values of current and T_on_. An increase in the current and T_on_ enhances the discharge energy during the sparking process. Thus, a large quantity of heat is transmitted in the gap between tool and work material [70]. Due to this heat, the amount of molten metal grows. Dielectric fluid was incapable of removing some of the eroded particles, which then became stuck on the work surface [35]. Those stuck particles then quenched and re-solidified by means of the formation of thick RLT. On the other hand, the initial increment in the value of T_off_ from 5 µs to 15 µs decreased the RLT. The probable reason behind this was the melting of work material. Also, a higher magnitude of T_off_ grants sufficient time to remove the eroded particles from the machining zone [34]. At the higher values of T_off_ from 15 µs to 25 µs, flushing time increases and the resolidified particles stick on machined surface. Ultimately, thicker RLT is formed.

### 3.6. Optimization Using PVS Algorithm

During the implementation of the PVS algorithm, all outcomes were considered as positive digits. The device bounds followed for the implementation of optimization were (Current): 2 A ≤ Current ≥ 6 A; (T_off_): 5 µs ≤ T_off_ ≥ 25 µs; (T_on_): 25 µs ≤ T_on_ ≥ 75 µs. Higher value of MRR, and lower values in the case of SR and RLT, were taken into account during individual response optimization. Table 7 depicted the obtained results and found the highest MRR of 2.6433 g/min, and the lowest SR and RLT of 3.45 µm and 10.88 µm, respectively. The obtained results of individual responses has not shown good agreement for remaining responses. This shows conflicting levels of machining variables. In such situations, generation of Pareto fronts can resolve these disputes. Pareto fronts offers trade-offs among such contradictory responses and give a choice to the user to select any point on the front as per the requirement.

**Figure 9:**
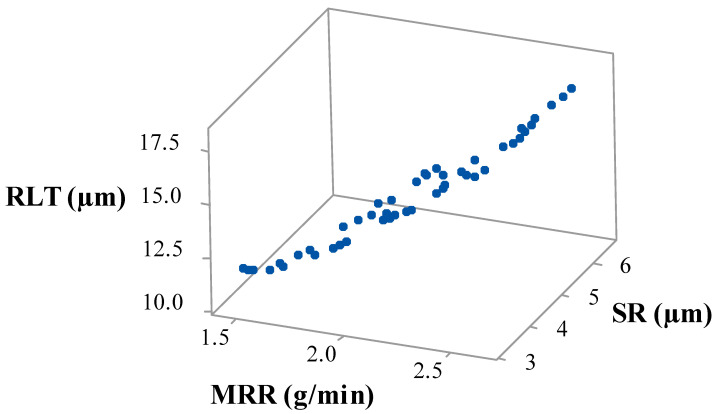
Pareto plot of MRR vs SR vs RLT.

The passing vehicle search (PVS) method was used to perform simultaneous optimization of specified output variables. Non-dominant Pareto points were generated. A 3D Pareto graph for al output variables can be seen from Figure 9. To obtain the desired optimum Pareto points, 10,000 evolution functions were utilized and 50 Pareto points were generated, data which provide for a unique solution. All points on the graph had their respective machining variables. Thus, an operator will have multiple options, and a selection will be made as per the necessary values of output variables. The nature of the Pareto graph clearly depicted that escalation was also leading to the higher values of SR and RLT. Validation experiments were performed to validate the results of the PVS algorithm. A negligible error was found in between the predicted and recorded values. This clearly reveals the acceptability of the generated model and PVS technique.

### 3.7. Influence of MWCNTs on MRR, SR, and RLT

Unique properties of MWCNTs, such as excellent mechanical and electrical properties, enhance both the machining performance and surface quality. When MWCNTs are combined with a dielectric fluid, their unique features such as high thermal conductivity and enlarged strength can affect the thermo-electrical properties of the dielectric medium, potentially improving the WEDM performance [76]. The election of a proper amount of powder concentration plays a key role in performance. In our recently concluded study for nickel-based superalloys (Nitinol), amount of 1 g/L MWCNT has shown enhancement in MRR and SR by 75.42% and 19.15%, respectively. RLT was also observed to be lowest for 1 g/L amount of MWCNT in comparison with the other concentrations. Therefore, 1 g/L MWCNT amount has been considered in current study to identify the influence of MWCNTs on MRR, SR, and RLT. For this purpose, a case study was employed which considers an equal weightage to all the output measures. A multi-objective optimization approach has been employed by giving identical weights to all performance measures. The generated equation for this scenario was represented as below (refer Equation (5)).

(5) $$\mathrm{Obj}\;=w_{1}\cdot \left(\mathrm{MRR}\right)+w_{2}\cdot \left(\mathrm{SR}\right)+w_{3}\cdot \left(\mathrm{RLT}\right)$$

Multi-response objective yielded optimized levels of MRR, SR, and RLT as 1.9801 g/min, 4.38 µm, and 13.11, respectively, at current of 4 A, T_off_ of 11 µs, T_on_ of 31 µs. 

Other trials have been carried with the same parameters, and 1 g/L MWCNT amount has been added to dielectric fluid to identify the influence of MWCNTs on MRR, SR, and RLT. Table 8 shows the comparison of the obtained results with both the experimental trials. As discussed earlier, conventional WEDM (without a MWCNT amount) has obtained optimized values of MRR, SR, and RLT as 1.9801 g/min, 4.38 µm, and 13.11, respectively, at current of 4 A, T_off_ of 11 µs, T_on_ of 31 µs. Conversely, the addition of MWCNT at 1 g/L amount has given the optimized values of MRR, SR, and RLT as 3.2811 g/min, 2.16 µm, and 7.74 µm, respectively. It concludes that the addition of MWCNT at 1 g/L has improved the performance of MRR, SR, and RLT by 65.70%, 50.68%, and 40.96%, respectively. All these improved responses to powder-mixed WEDM have given even more improvements in comparison to their single-objective optimization results. Distribution of MWCNTs into dielectric enhances the thermal conductivity and spark occurrences [47]. Thus, erosion of particles from workpiece intensified and enlarged the MRR. The addition of nano-powder magnified the inter-electrode gap and developed tiny craters owing to the rise of heat dissipation and reduction of plasma heat flux [41,77]. Inclusion of MWCNT amount has improved removal of debris from machining zone. Higher removal of eroded particles thus formed tiny ridges and has obtained better surface features. Suspended particles of MWCNTs reduces the insulation capability and also enhances the inter-electrode gap [78]. Owing to these facts, RLT reduces with addition of MWCNT particles. This shows that MWCNTs at 1 g/L improves all the response variables at larger extend and found to be suitable for WEDM machining of Waspaloy.

### 3.8. Influence of MWCNTs on Surface Integrity

**Figure 10:**
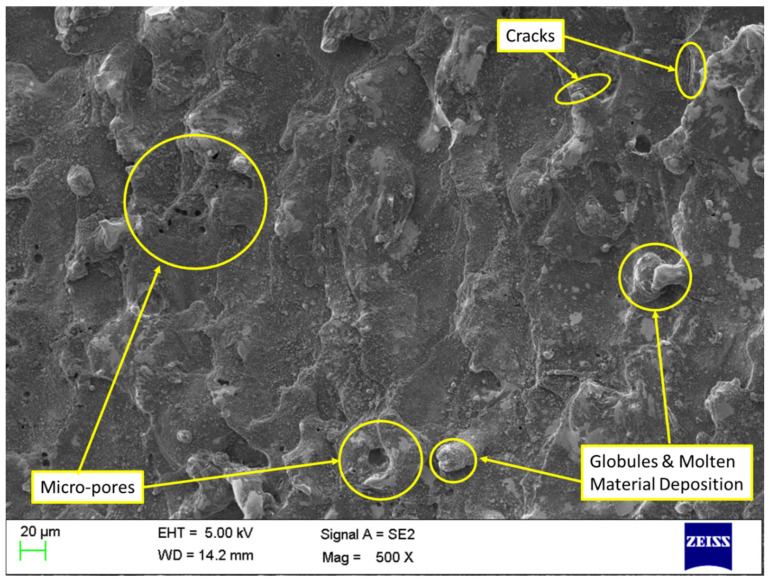
Analysis of machined surface for conventional WEDM (without MWCNTs).

**Figure 11:**
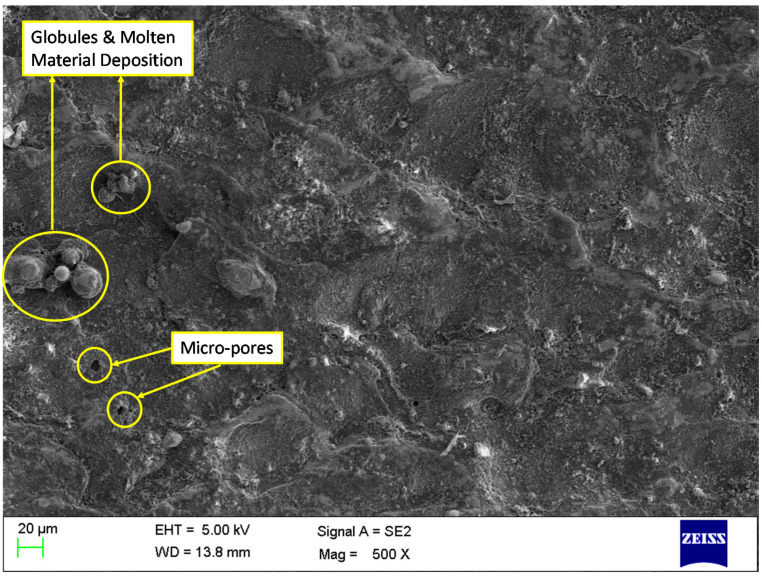
Analysis of machined surface for NPMWEDM (addition of MWCNTs at 1 g/L).

Effect of MWCNTs on MRR, SR, and RLT has shown outstanding improvements. However, study pertaining to the impact of MWCNT on surface morphology is necessary. To understand this, SEM was utilized to assess surface defects formed on the machined surfaces of conventional WEDM and nano-powder-mixed WEDM. Machined samples obtained as per Table 8 has been considered in this study. Machined surfaces produced by conventional WEDM (at current of 4 A, T_off_ of 11 µs, T_on_ of 31 µs, and MWCNT of 0 g/L) and nano-powder-mixed WEDM (at current of 4 A, T_off_ of 11 µs, T_on_ of 31 µs, and MWCNT of 1 g/L) were shown in Figure 10 and 11, respectively. Analyzing the surface produced in Figure 10 discloses a considerable occurrence of micro-pores, a greater layer admission (development of globules), and the existence of micro-cracks. On the other hand, the surface morphology achieved at MWCNTs at 1 g/L, as shown in Figure 11, demonstrated a considerable progress in surface defects, i.e., substantial drop in micro-pores, globules, and almost complete removal of micro-cracks. Consistent occurrences of sparking among tool and workpiece due to nano-powder has resulted in the almost complete removal of micro-cracks [56,78]. Suspended MWCNT particles expand the machining zone (gap in between the work material and tool) and form tiny craters on the machined surface [53,79]. Also, MWCNT particles further improves the flushing of debris particles. This further results in improvements in surface quality. Additionally, high thermal conductivity of MWCNTs increases the heat dissipation and thereby decreases plasma heat flux [45,80]. Owing to these reasons, the addition MWCNTs in dielectric fluid largely diminishes the surface defects in the form of globules, and micro-pores. Thus, the obtained SEM graph established that use of MWCNT amount of 1 g/L has improved the quality of machined parts by means of decreasing the surface defects.

## 4. Conclusions

The current study attempts to establish techniques for the precise machining of Waspaloy by using WEDM process. The effect of MWCNTs, along with optimization through the use of a PVS algorithm, has been determined by considering current, T_off_, and T_on_ as WEDM parameters on MRR, SR, and RLT of Waspaloy. BBD of RSM was employed to generate the experimental matrix. A summary of the obtained results can be drawn as below:

- ANOVA for MRR showed that all model terms were having a noteworthy impact on MRR. With respect to MRR, all machining variables were having a significant effect. Current was found to have the higher impact on MRR followed, by T_off_ and T_on_.
- A similar conclusion can be made for SR, and RLT of ANOVA, for which all the model terms were observed to have substantial impact. For SR, all machining variables had a significant effect, with a large involvement of T_on_, followed by current and T_off_. On the other hand, RLT, current and T_on_ had significant effect, with a large impact for current, and T_off_ was noticed to be a non-significant factor.
- Lack of fit was found to be non-significant for all responses, suggesting the suitability of developed model terms to forecast the future outcomes. Additionally, minor difference between R^2^ values showed the suitability of the obtained results and the proposed model.
- Verification of four tests of residual plots for all responses signified good ANOVA results and satisfied the necessary conditions for ANOVA.
- A PVS algorithm was implemented for finding the optimum solution of various responses. Individual response optimization has produced highest MRR of 2.6433 g/min and lowest SR and RLT of 3.45 µm and 10.88 µm, respectively.
- Non-dominant Pareto points were produced from PVS algorithm which has given independent and unique solutions. Minor acceptable deviation was recorded among the anticipated and recorded values. This clearly reveals the acceptability of the generated model and PVS technique.
- Machining performance was enhanced by adding MWCNTs at 1 g/L. Accumulation of MWCNTs at 1 g/L has improved the performance of MRR, SR, and RLT by 65.70%, 50.68%, and 40.96%, respectively.
- A reduction of globules of debris, micro-pores and micro-crack free surface, melted material deposition, was observed in terms of surface morphology, wherein 1 g/L MWCNTs was used.

## Figures and Tables

**Table 1:** Composition by wt. % for Waspaloy.

Element	Ni	Cr	Co	Mo	Ti	Al	Fe	Si	C	Zr	Cu
Wt%	58.97	20.94	13.09	5.74	2.79	1.24	0.78	0.066	0.062	0.058	0.03

**Table 2:** WEDM input variables.

Process Parameter	Levels/Values
Pulse-on time, T_on_	25; 50; 75
Pulse-off time, T_off_	7; 14; 21
Discharge current	2; 4; 6
Nano-Powder	MWCNT
Nano powder-size (nm)	10–20

**Table 3:** Experimental plan as per RSM of BBD and readings of MRR, SR, and RLT.

Exp. No.	Current (A)	T_off_ (µs)	T_on_ (µs)	MRR (g/min)	SR (µm)	RLT (µm)
1	4	15	50	1.8959	4.82	13.02
2	6	15	25	2.3941	4.75	16.00
3	2	15	75	1.8669	5.82	12.50
4	4	5	75	2.1421	6.34	14.32
5	6	15	75	2.5227	6.40	16.85
6	2	5	50	1.7393	5.38	11.65
7	2	25	50	1.4926	4.09	12.80
8	6	25	50	2.3125	5.57	15.47
9	4	25	25	1.7543	3.80	12.20
10	2	15	25	1.6455	4.18	11.02
11	6	5	50	2.4918	5.44	16.70
12	4	15	50	1.8727	4.75	12.45
13	4	15	50	1.9018	4.69	12.72
14	4	5	25	2.1803	4.42	14.62
16	4	25	75	2.1881	5.88	16.97

**Table 4:** ANOVA for MRR.

Source	DoF	SS	MS	F	P	Significance
Model	7	1.4034	0.2004	179.56	0.000	#
Linear	3	1.2582	0.4194	375.65	0.000	#
Current	1	1.1075	1.1075	991.98	0.000	#
T_off_	1	0.0812	0.0812	72.72	0.000	#
T_on_	1	0.0695	0.0695	62.24	0.000	#
Square	3	0.0894	0.0298	26.70	0.000	#
Current × Current	1	0.0236	0.0236	21.17	0.002	#
T_off_ × T_off_	1	0.0055	0.0055	5.00	0.060	*
T_on_ × T_on_	1	0.0694	0.0694	62.21	0.000	#
2-Way Interaction	1	0.0557	0.0557	49.91	0.000	#
T_off_ × T_on_	1	0.0557	0.0557	49.91	0.000	#
Error	7	0.0078	0.0011			#
Lack of fit	5	0.0073	0.0014	6.17000	0.14500	*
Pure error	2	0.0004	0.0002			
Total	14	0.5010				

R-Sq. = 99.45%; R-Sq. (Adj.) = 98.89%, # = Significant term; * = Insignificant term.

**Table 5:** ANOVA for SR.

Source	DoF	SS	MS	F	P	Significance
Model	6	9.1406	1.5234	92.11	0.000	#
Linear	3	8.1674	2.7224	164.61	0.000	#
Current	1	0.8971	0.8971	54.24	0.000	#
T_off_	1	0.6336	0.6336	38.31	0.000	#
T_on_	1	6.6366	6.6366	401.28	0.000	#
Square	2	0.4655	0.2328	14.08	0.002	#
Current × Current	1	0.2608	0.2608	15.77	0.004	#
T_on_ × T_on_	1	0.2379	0.2379	14.39	0.005	#
2-Way Interaction	1	0.5076	0.5076	30.69	0.001	#
Current × T_off_	1	0.5076	0.5076	30.69	0.001	#
Error	8	0.1323	0.0165			
Lack of fit	6	0.1232	0.0205	4.52	0.192	*
Pure error	2	0.0090	0.0045			
Total	14	9.2729				
Model	6	9.1406	1.5234	92.11	0.000	#

R-Sq. = 99.45%; R-Sq. (Adj.) = 98.89%, # = Significant term; * = Insignificant term.

**Table 6:** ANOVA for RLT.

Source	DoF	SS	MS	F	P	Significance
Model	8	56.0144	7.0018	35.84	0.000	#
Linear	3	42.1206	14.0402	71.87	0.000	#
Current	1	36.3378	36.3378	186.01	0.000	#
T_off_	1	0.0028	0.0028	0.01	0.908	*
T_on_	1	5.7800	5.7800	29.59	0.002	#
Square	3	6.0447	2.0149	10.31	0.009	#
Current × Current	1	0.8964	0.8964	4.59	0.076	*
T_off_ × T_off_	1	3.1949	3.1949	16.35	0.007	#
T_on_ × T_on_	1	2.7800	2.7800	14.23	0.009	#
2-Way Interaction	2	7.4891	3.9245	20.09	0.002	#
Current × T_off_	1	1.4102	1.4102	7.22	0.036	#
T_off_ × T_on_	1	6.4389	6.4389	32.96	0.001	#
Error	6	1.1720	0.1954			
Lack of fit	4	1.0067	0.2517	3.04	0.262	*
Pure error	2	0.1654	0.0827			
Total	14	57.1865				

R-Sq. = 99.45%; R-Sq. (Adj.) = 98.89%; # = Significant term; * = Insignificant term.

**Table 7:** Single Objective Optimization using PVS algorithm.

Objective Function	Design Variables			Objective Function		
	Current	T_on_	T_off_	MRR	SR	RLT
**Maximum MRR**	6	25	5	2.6433	4.68	18.14
**Minimum SR**	2	25	25	1.4617	3.45	11.38
**Minimum RLT**	2	38	15	1.5841	4.36	10.88

**Table 8:** Influence of MWCNT on MRR, SR, and RLT.

Condition	WEDM Variables	Response Variables
Conventional WEDM (Without MWCNT amount)	Current = 4 A T_off_ = 11 µs T_on_ = 31 µs MWCNT = 0 g/L	MRR = 1.9801 g/min SR = 4.38 µm RLT = 13.11 µm
MWCNTs mixed WEDM (Addition of MWCNTs at 1 g/L)	Current = 4 A T_off_ = 11 µs T_on_ = 31 µs MWCNT = 1 g/L	MRR = 3.2811 g/min SR = 2.16 µm RLT = 7.74 µm

## Data Availability

Data presented in this study are available in this article.
